# Tyrosine Kinase Inhibitor Treatment of a Patient with Chronic Myeloid Leukemia and Congenital Thrombophilia

**DOI:** 10.3390/hematolrep17050047

**Published:** 2025-09-12

**Authors:** Carol Herrera-Hernández, Adrián Segura-Diaz, Ruth Stuckey, Juan Francisco López-Rodríguez, María Teresa Gómez-Casares

**Affiliations:** 1 Hematology Department, Hospital Universitario de Gran Canaria Dr. Negrín, 35010 Las Palmas, Spain; karolh098@gmail.com (C.H.-H.); asegdia@gobiernodecanarias.org (A.S.-D.); jfloprodr@gobiernodecanarias.org (J.F.L.-R.); mgomcasf@gobiernodecanarias.org (M.T.G.-C.); 2 Department of Medical Sciences, University of Las Palmas de Gran Canaria, 35010 Las Palmas, Spain

**Keywords:** tyrosine kinase inhibitor (TKI), chronic myeloid leukemia, cardiovascular risk, congenital thrombophilia, protein S, anticoagulation

## Abstract

**Background and Clinical Significance:** Chronic Myeloid Leukemia (CML) management has been revolutionized by tyrosine kinase inhibitors (TKIs), though cardiovascular and thrombotic complications remain a concern, especially in patients with underlying risk factors. Inherited thrombophilia, including protein S deficiency and Factor V Leiden mutation, poses a substantial risk for venous thromboembolism (VTE). Managing CML in patients with such prothrombotic predispositions presents complex therapeutic challenges, particularly in selecting an appropriate TKI and managing anticoagulation. **Case Presentation:** A 33-year-old woman with congenital thrombophilia (type I protein S deficiency and heterozygous Factor V Leiden mutation) and a history of VTE on long-term anticoagulation with acenocoumarol presented with CML. She exhibited primary resistance to first-line imatinib and poor tolerance with suboptimal response to second-line bosutinib. Third-line treatment with asciminib led to a rapid and sustained major molecular response (MR4.5) without bleeding or thrombotic complications. **Conclusions:** This case highlights the importance of individualized, multidisciplinary management in CML patients with coexisting thrombophilia. Asciminib, with its favorable cardiovascular safety profile, represents a promising therapeutic option in high-risk patients where other TKIs may be contraindicated due to resistance, intolerance, or thrombotic risk.

## 1. Introduction

Chronic Myeloid Leukemia (CML) is a myeloproliferative neoplasm driven by the BCR::ABL1 fusion gene, which encodes a constitutively active tyrosine kinase. The introduction of tyrosine kinase inhibitors (TKIs) has dramatically improved the prognosis of CML, transforming it from a life-threatening condition into a manageable chronic disease. However, despite their efficacy, the long-term use of TKIs has been associated with a range of significant cardiovascular side effects that can limit their long-term use and impact patient health [1].

While the first-generation TKI, imatinib, is generally well-tolerated, second- and third-generation TKIs exhibit varying cardiovascular toxicity profiles. The incidence of cardiac and arteriothrombotic adverse events are particularly high for ponatinib and nilotinib, with vascular events also frequent in patients treated with dasatinib [2,3]. The CV and thrombotic risk associated with TKIs vary depending on the specific agent used. For example, dasatinib is linked to pulmonary hypertension, nilotinib to coronary atherothrombotic, cerebrovascular, and peripheral arterial events, and bosutinib to arterial hypertension [4]. Ponatinib, a third-generation TKI, is notably associated with both arterial and venous occlusive events, including life-threatening thromboembolic events. Alarmingly, up to 50% of CML patients may succumb not to the disease itself, but to cardiovascular complications or secondary malignancies [5], underscoring the importance of understanding the cardiovascular impacts of these therapies.

Inherited thrombophilia, characterized by a genetic predisposition to venous thromboembolism (VTE), includes deficiencies of natural anticoagulants such as protein S, protein C, and antithrombin, as well as gain-of-function mutations like Factor V Leiden and the prothrombin G20210A variant [3]. Protein S is a vitamin K–dependent protease that circulates in plasma at low concentrations and serves a crucial role in the regulation of coagulation. In normal circumstances, the anticoagulant proteins keep the blood in a liquid, non-thrombotic state. In circulation, approximately 40% of protein S is free, and about 60% is in a high-affinity complex with the complement regulatory factor C4b-binding protein (C4BP). Protein S serves as a cofactor for activated protein C (APC), enhancing APC-mediated degradation of activated factors V and VIII, and also supports tissue factor pathway inhibitor (TFPI) activity, thereby regulating thrombin generation [6].

The *PROS1* gene, located on chromosome 3q11.1, encodes protein S. The gene spans approximately 80 kilobases and contains 15 exons. Importantly, *PROS1* is highly polymorphic, with more than 200 pathogenic variants described. These include missense and nonsense mutations, splice-site defects, insertions, deletions, and large genomic rearrangements [7]. However, the sequencing of exons and splice junctions identifies pathogenic mutations in only ~50% of affected families, while larger deletions and insertions account for approximately 30% of the remaining cases [6,7].

Loss-of-function mutations in *PROS1* underlie hereditary protein S deficiency, which follows an autosomal dominant inheritance pattern. Haploinsufficiency is the predominant mechanism: a single functional copy of the gene is insufficient to maintain normal plasma protein S levels, predisposing affected individuals to venous thromboembolism (VTE). In type I deficiency, both free and total protein S are reduced due to decreased synthesis or stability; type II deficiency results from qualitative defects affecting APC cofactor activity; and type III deficiency is characterized by reduced free protein S despite normal total levels, often due to altered binding to C4b-binding protein [6].

According to the International Society on Thrombosis and Haemostasis (ISTH), the recommended approach to diagnosing protein S deficiency is a free protein S antigen assay. If the free protein S antigen level is abnormal, additional testing—protein S activity and total antigen levels—should be conducted to determine the deficiency subtype (classified as Type I: decreased free and total protein S; Type II: functional deficiency but normal protein S levels; Type III: low free protein S but normal concentration of total protein S). Notably, these assays should be avoided during pregnancy, hormone therapy, or treatment with vitamin K antagonists (VKAs) or direct oral anticoagulants (DOACs) [8].

The increased risk of thrombotic events associated with protein S deficiency (with an odds ratio of 5.4 for VTE) is further amplified in patients with coexisting Factor V Leiden mutation, which is known to confer resistance to activated protein C, further predisposing individuals to thrombosis [9]. Patients with inherited thrombophilia may require long-term anticoagulation as VTE prophylaxis.

In this case report, we discuss the clinical course, therapeutic decisions, and implications for managing a patient with inherited thrombophilia characterized by protein S deficiency (type I) and heterozygous Factor V Leiden mutation who is diagnosed with CML.

## 2. Case Presentation

This is the case of a 33-year-old woman with a history of thrombophilia due to protein S deficiency type I (with decreased levels of both total and free protein S antigen) and heterozygous factor V Leiden mutation. She was on long-term anticoagulation with acenocoumarol (Sintrom) due to prior venous thromboembolic events, including deep vein thrombosis (DVT) in 2018 associated with inflammatory bowel disease (IBD).

The patient presented to the emergency department in September 2020 with acute abdominal pain of 4 days’ evolution and marked mucocutaneous pallor, with diffuse abdominal tenderness on examination. Laboratory testing revealed severe leukocytosis (285,000/µL), anemia (hemoglobin 9.75 g/dL), elevated C-reactive protein (62.8 mg/L), and profound coagulation abnormalities while on anticoagulation therapy (INR 5.68; prothrombin time 67.7 s; aPTT 50.5 s). These findings raised suspicion of significant intra-abdominal bleeding. Abdominal CT confirmed the presence of hemoperitoneum without clear origin. A standard preoperative evaluation including chest radiography and electrocardiogram was also performed, with normal results.

Given the combination of acute anemia and severe coagulopathy, urgent surgical exploration was indicated, and intraoperatively the bleeding source was identified as a spontaneous rupture of an ovarian cyst with more than 3 L of hemoperitoneum. The severity of the hemorrhage was attributed to the underlying prothrombotic disorder and chronic anticoagulation, which transformed an otherwise often self-limited event into a life-threatening complication.

Blood tests carried out after surgery showed continued leukocytosis (162,000/µL) and anemia (hemoglobin 9.66 g/dL), and splenomegaly (15.9 cm). Given the hematological abnormalities, a myeloproliferative disorder was suspected. Laboratory tests revealed BCR::ABL1 positivity in peripheral blood by PCR (p210), confirming the diagnosis of chronic-phase CML. The patient was stratified as low-risk using the Sokal, EUTOS, ELTS, and Hasford scores.

The patient was initiated on imatinib (400 mg daily) in September 2020. After three and six months of TKI, she demonstrated a suboptimal response, with a BCR::ABL1^IS^ level of 11% and 5.6%, respectively. Tyrosine kinase domain mutation analysis failed to detect any mutations associated with resistance, and the patient’s adherence to therapy was confirmed. Primary resistance to imatinib was suspected when her transcripts increased to 17% in April 2021 (Table 1), prompting the decision to switch to second-line therapy.

Bosutinib (400 mg daily) was selected for second-line treatment based on its favorable CV risk profile, considering the patient’s high thrombotic risk, with the exclusion of dasatinib and nilotinib due to their known procoagulant profiles. However, bosutinib was poorly tolerated due to gastrointestinal side effects, and the patient continued to show a suboptimal response after three (3.77% transcripts), six (1.69% transcripts) and nine months (1.26% transcripts) of bosutinib treatment (Table 1). Given the inadequate molecular response and prolonged gastrointestinal intolerance, the TKI was switched to third-line asciminib (40 mg twice daily) in April 2022.

The transition to asciminib led to a major molecular response (MMR) within two months (BCR::ABL1^IS^ 0.029%, Table 1). The response deepened to MR4 after one year of treatment, by August 2023, and to MR4.5 in November 2023, with good tolerance.

**Figure 1:**
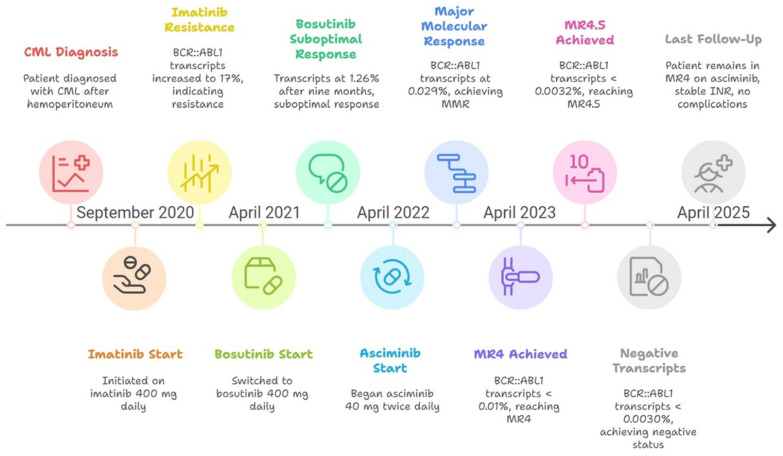
Timeline of the clinical case. CML: chronic myeloid leukemia; INR: international normalized ratio; MR: molecular response; MMR: major molecular response.

At last follow-up (April 2025), the patient remained in MR4 on 40 mg BID asciminib. No bleeding complications or thrombotic events were experienced during the course of therapy with three different TKIs (timeline of case, Figure 1). The patient’s INR has remained stable (2–3) throughout her follow-up, since the hemoperitoneum event and consequential CML diagnosis.

## 3. Discussion

To the best of our knowledge, this is the first reported case of CML in a patient with hereditary protein S deficiency successfully treated with asciminib. While previous reports have described challenges in managing CML in patients with other thrombophilic conditions, such as factor V Leiden or antiphospholipid syndrome, the coexistence of protein S deficiency presents unique therapeutic considerations. Protein S deficiency is associated with a particularly high risk of recurrent venous thromboembolism, and the need to balance lifelong anticoagulation with CML therapy creates a distinctive clinical scenario. The favorable outcome in our patient underscores the feasibility and safety of asciminib in a high-risk patient receiving long-term anticoagulation.

This unique clinical scenario presented significant challenges in selecting an appropriate TKI, given the patient’s preexisting thrombotic risk since some TKIs have been implicated in endothelial dysfunction, prothrombotic states, and metabolic disturbances, which may exacerbate an underlying thrombophilic condition [10]. The presence of both inherited thrombophilia and CML necessitated a careful balance between achieving optimal leukemia control while mitigating thrombotic complications. This heightened thrombotic potential necessitated a tailored treatment strategy for our patient, both in terms of TKI selection and anticoagulation management.

Protein S deficiency is a well-established risk factor for VTE, with affected individuals exhibiting a 5- to 10-fold increased risk of thrombosis and an annual recurrence risk of 6% to 10%, placing them at a persistently high risk of vascular events [6,11,12]. Our patient initially presented with hemoperitoneum secondary to the spontaneous rupture of an ovarian cyst, with intraoperative findings of >3 L of intra-abdominal bleeding. While such events are typically self-limiting, in this case the coexistence of hereditary protein S deficiency and anticoagulation therapy markedly increased the risk of severe hemorrhage, ultimately necessitating urgent surgical intervention. This episode highlights how common gynecological or otherwise benign events can be significantly complicated in patients with underlying thrombophilia on chronic anticoagulation. Moreover, thrombotic risk in such patients may be further exacerbated by coexisting CML and its treatment.

The vascular toxicity of TKIs is largely influenced by the inhibitors’ selectivity for BCR::ABL1 and their off-target effects on kinases involved in vascular homeostasis, with a positive correlation between cardiotoxicity and the total number of kinases inhibited [13]. While imatinib and bosutinib have relatively favorable CV safety profiles, other TKIs such as dasatinib, nilotinib, and ponatinib can increase thrombotic risk due to their procoagulant and cardiovascular effects. Asciminib, a novel STAMP inhibitor, was ultimately chosen due to its unique mechanism of action and its promising CV safety profile [14]. While experience with asciminib is much shorter compared to the other five TKIs approved for CML treatment, data from pivotal trials and real-world studies suggest a lower incidence of CV events [15,16]. In our patient, a sequential TKI approach ultimately led to an optimal and sustained molecular response with asciminib, with no thrombotic or bleeding complications, further supporting its efficacy and safety in this high-risk patient.

The American College of Chest Physicians (ACCP) 2016 guidelines on antithrombotic therapy for VTE favor DOACs over VKAs for most patients with VTE [17]. However, specific recommendations on the treatment of VTE in patients with inherited thrombophilia are lacking, and there are currently no standardized guidelines for anticoagulation in patients receiving TKIs with concomitant pathologies. The decision to continue long-term anticoagulation with acenocoumarol (a VKA) in our patient was based on the underlying hereditary thrombophilia and prior thrombotic events. While DOACs are now widely used, evidence for their safety and efficacy in protein S deficiency remains limited, and concerns about potential drug–drug interactions with TKIs also influenced our choice.

Notably, asciminib, unlike ATP-competitive TKIs such as imatinib, bosutinib, nilotinib, or dasatinib, has a distinct mechanism of action as a STAMP inhibitor. This allosteric inhibition confers a more selective pharmacologic profile, which may reduce the risk of off-target effects and drug–drug interactions [18]. According to the European Medicines Agency (EMA), asciminib is classified as a reversible inhibitor of several CYP enzymes in vitro, including CYP3A4/5, CYP2C8, and CYP2C9 [19]. These findings are based on studies in human liver microsomes, which showed inhibition at clinically relevant concentrations, although the degree of inhibition was modest. However, clinical pharmacokinetic studies suggest that asciminib does not significantly alter the plasma levels of CYP3A4 or CYP2C9 substrates, indicating that in vivo inhibition is limited [20,21]. This is clinically relevant, as CYP2C9 plays a role in the metabolism of several anticoagulants, including the VKAs aceoncoumarol and the R-isomer of warfarin, while CYP3A4 is involved in the metabolism of certain DOACs, like rivaroxaban and apixaban. Indeed, inhibition of CYP3A4 by other TKIs has been associated with altered plasma concentrations of co-administered drugs, potentially leading to bleeding complications or reduced anticoagulant efficacy [22]. Since asciminib shows minimal in vivo inhibition, it is unlikely to significantly affect the plasma levels of these anticoagulants and thus appears to have a favorable interaction profile with VKAs such as acenocoumarol. Nevertheless, caution is still warranted, especially in polypharmacy settings since no direct studies have evaluated asciminib’s interaction with acenocoumarol specifically. Importantly, according to a recent systematic review, there is still very little clinical data on the safety of combining TKIs and anticoagulants, representing an unmet research need [23].

Furthermore, published data indicate that asciminib exhibits a predictable pharmacokinetic profile and a lower incidence of clinically relevant drug–drug interactions compared to ATP-competitive TKIs. These properties, combined with the patient’s thrombophilia and prior thrombotic history, provided additional rationale for maintaining stable anticoagulation with a VKA rather than switching to a DOAC. In the absence of standardized guidelines, anticoagulant management in patients with inherited thrombophilia receiving TKIs must remain individualized, taking into account bleeding risk, drug metabolism pathways, and the specific TKI selected.

The European Society of Cardiology (ESC) recommends baseline CV risk assessment in all cancer patients receiving potentially cardiotoxic agents, including TKIs [24]. In high-risk patients, early cardiology assessment and structured follow-up may be beneficial. While there are no specific surveillance protocols for vascular events in inherited thrombophilia patients on TKIs, echocardiographic and vascular assessments, such as the ankle-brachial index (ABI) every 6–12 months, may be warranted [25]. Clinicians should be especially aware of other acquired or transient risk factors that can aggravate vascular event risk and may require additional management considerations. Special attention should be given to the use of oral contraceptives and during pregnancy (as both significantly reduce PS levels), as well as surgery or hospitalization [26]. For our 33-year-old high-risk female patient, given the additional thrombotic risk conferred by protein S deficiency, fertility and pregnancy considerations were discussed. Protein S deficiency is associated with pregnancy complications, including increased risk of venous thromboembolism and adverse obstetric outcomes. Therefore, counseling regarding contraceptive options and careful pregnancy planning is essential in such patients. In our case, contraceptive counseling was provided, and the potential risks of pregnancy during CML therapy, particularly while on asciminib, were reviewed with the patient.

Our report underscores the importance of individualized treatment approaches in patients with concurrent thrombophilia and CML. Balancing TKI therapy with long-term anticoagulation can be a challenge, considering both the prothrombotic risk and potential bleeding complications. TKI options are particularly limited for patients with inherited thrombophilia, especially in cases of TKI resistance or intolerance, as described in this case. Close monitoring for vascular complication and an individualized anticoagulation strategy is recommended for CML patients, especially in those with additional prothrombotic risk factors.

## 4. Conclusions

The interplay between CML, inherited thrombophilia, and TKI therapy presents a complex clinical challenge requiring a multidisciplinary and personalized approach. Asciminib is a promising option for achieving deep molecular responses while minimizing cardiovascular and coagulopathy-related complications. Given the lack of standardized protocols for managing thrombophilia in CML, individualized therapeutic strategies remain paramount to optimizing patient outcomes.

## Figures and Tables

**Table 1:** Molecular response of the patient. First-line tyrosine kinase inhibitor treatment commenced with imatinib three days after diagnosis. IS: International scale.

Disease Evolution	Inhibitor	Date	*BCR::ABL1/ABL1* ^IS^
Diagnosis		20 September 2020	74%
First-line therapy	Imatinib	23 September 2020	74%
		20 November 2020	31%
		08 January 2020	11%
		18 February 2021	5.61%
		24 April 2021	17%
Second-line therapy	Bosutinib	03 September 2021	3.77%
		11 October 2021	3.61%
		25 November 2021	1.69%
		05 January 2022	0.7%
		03 March 2022	1.26%
Third-line therapy	Asciminib	06 July 2022	0.049%
		10 August 2022	0.029%
		10 January 2023	0.017%
		21 April 2023	0.016%
		10 August 2023	0.012%
		07 November 2023	0.0015%
		04 February 2024	0.0096%
		31 October 2024	0.0044%
		23 January 2025	Negative *
		15 April 2025	0.0031%

* Negative: transcripts < 0.0030%.

## Data Availability

The original contributions presented in this study are included in the article. Further inquiries can be directed to the corresponding author(s).
